# The Influence of Hazelnut Milk Fortification on Quality Attributes of Probiotic Yogurt

**DOI:** 10.1002/fsn3.70235

**Published:** 2025-05-04

**Authors:** Selin Kalkan, Kübra Incekara, Mustafa Remzi Otağ, Emel Unal Turhan

**Affiliations:** ^1^ Faculty of Engineering, Department of Food Engineering University of Giresun Giresun Turkey; ^2^ Faculty of Kadirli Applied Sciences, Department of Food Technology University of Osmaniye Korkut Ata Osmaniye Turkey

**Keywords:** bioactivity, hazelnut milk, probiotic, textural analysis, yogurt

## Abstract

This study evaluated probiotic yogurt fortified with varying proportions of hazelnut milk (10%, 20%, 30%, 40%, and 50%) for its physicochemical, textural, microbiological, and bioactive properties during refrigerated storage. The incorporation of hazelnut milk and the storage duration significantly influenced the overall quality attributes of yogurt. Increasing the proportion of hazelnut milk from 0% to 50% significantly influenced pH levels (rising from 4.22 to 5.16), likely due to the reduced buffering capacity of plant‐based milk. Yogurt samples produced with 50% hazelnut milk exhibited the highest dry matter (15.22%), protein content (4.11%), and total phenolic content, along with the strongest DPPH radical scavenging activity. Total phenolic content ranged from 30.12 to 155.29 mg/L, while antioxidant activity increased from 7.27% to 46.59% with higher hazelnut milk concentrations, indicating enhanced bioactive properties. Microbiological analysis revealed improved probiotic viability at higher hazelnut milk levels (30%–50%), with total lactic acid bacteria counts increasing from 7.38 to 8.58 log CFU/g during storage. Microstructural analysis showed that increased hazelnut milk content resulted in a more homogeneous and smoother protein network. The values for hardness (from 0.43 to 1.01), consistency (from 4.22 to 10.63), internal stickiness (from −0.10 to −0.46), and viscosity (from −0.03 to −0.51) of the yogurt samples changed significantly depending on the proportion of hazelnut milk. Textural parameters—including hardness (0.43–1.01), consistency (4.22–10.63), internal stickiness (−0.10 to −0.46), and viscosity index (−0.03 to −0.51)—also varied significantly with hazelnut milk addition, indicating firmer, thicker, and less adhesive yogurts with modified flow properties. Among all formulations, the 50% hazelnut milk‐enriched sample demonstrated the most favorable overall quality. These findings suggest that hazelnut milk fortification can effectively improve the functional, structural, and microbiological quality of probiotic yogurt, although its high pH and lower acidity levels may influence fermentation dynamics and shelf stability.

## Graphical abstract

This study investigated the effects of hazelnut milk fortification (10%–50%) on the quality of probiotic yogurt during refrigerated storage. Increasing hazelnut milk levels led to higher pH, dry matter, protein content, total phenolics, and antioxidant activity. Probiotic viability improved, and microstructure became smoother with higher hazelnut content. Textural properties also changed, resulting in firmer and thicker yogurt. The formulation with 50% hazelnut milk showed the best overall quality. However, elevated pH may influence fermentation and shelf stability.

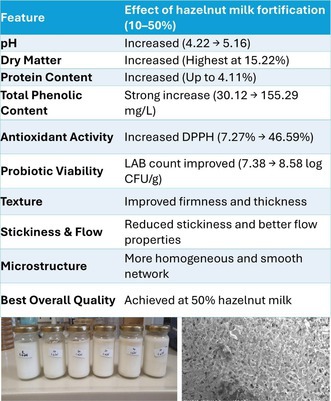

## 1 Introduction

Probiotics as functional food components are microbial food supplements that, when consumed in certain quantities, have positive effects on host health. Probiotics have been found to have beneficial effects such as addressing lactose intolerance, cancer, high cholesterol, and intestinal disorders caused by antibiotic use (Parvez et al. 2006; Michalak and Katarzyna 2016). The main probiotic foods are yogurt and fermented milk beverages. In recent years, probiotic cultures have been incorporated into the composition of fermented milk products in addition to the classic yogurt starters, providing extra physiological effects and nutritional value to the product (Canbulat and Ozcan 2015). Besides their nutritional properties, dairy and dairy products also hold special importance in preventing diseases. Regular consumption of probiotic fermented dairy products contributes to improving the microbial balance in the intestines, enhancing vitamin production, increasing protein digestion and mineral absorption, as well as boosting lactase production. Additionally, previous studies have highlighted their potential roles in strengthening the immune system, reducing the risk of colon cancer, lowering serum cholesterol levels, preventing diarrhea, inhibiting the growth of specific pathogenic bacteria, and exhibiting anti‐carcinogenic and anti‐allergenic effects (Shah 2007; Falah et al. 2021; Behbahani et al. 2023). Yogurt, among these products, is generally considered a good source of probiotics (Isanga and Zhang 2009). Interest in probiotic yogurts containing *Lactobacillus acidophilus* , *Bifidobacterium* species, and *L. casei* is steadily increasing. For realizing health benefits, the probiotic bacteria in yogurt must be live and present in high concentrations (typically ≥ 10^6^ CFU/g product). Moreover, to reach the target organs and systems, these bacteria should be able to pass through the digestive system alive (Shah 2007).

Fermented dairy products contain bioactive components and essential nutrients that are beneficial for human health. Therefore, it is reasonable as part of a healthy diet. It is recommended to consume dairy products. People who follow a vegan diet, are lactose intolerant or allergic to milk proteins do not consume milk. Plant‐based milk and dairy products have been developed for those who cannot consume animal milk. Animal welfare and environmental issues as a driving force in society have increased the consumption of plant‐based dairy products (Raikos et al. 2020). In the literature, plant milk is defined as “cereals, legumes or oilseeds, or water extracts of cereals that resemble the appearance of cow's milk” (Yilmaz‐Ersan and Topcuoglu 2022). These kinds of milk, which can be consumed by vegans, are suspensions containing plant substitutes and decomposed plant materials dissolved in water. These milk are considered as functional foods and nutraceuticals because they are rich in bioactive components such as minerals, vitamins, dietary fibers and antioxidants (Erk et al. 2019). Plant milks have lower protein content than animal milks and the amount and bioavailability of nutrients are generally lower. For example, vegetable milk does not contain sufficient amounts of nutrients such as essential amino acids, vitamin D, calcium, iodine and iron in the composition of milk (Yilmaz‐Ersan and Topcuoglu 2022). Dairy products such as fermented beverages, yogurt and cheese produced by adding probiotic microorganisms to milk obtained from plants are alternative foods for vegans and vegetarians. Examples of plant milk are oat milk, rice milk, soya milk, pea milk, potato milk, flax milk, poppy milk, almond milk, hazelnut milk, cashew milk, coconut milk, etc. From a nutritional point of view, soya milk is the best alternative vegetable milk to cow's milk. However, due to its bean‐like flavor and lack of some nutrients, the demand for almond milk has increased in recent years (Erk et al. 2019).

Although yogurt is nutritionally valuable due to its peptides, proteins, vitamins, and minerals, some important substances, such as bioactive and phytochemical components, are not typically found in yogurt. In recent years, due to increasing consumer demand for nutritional and healthy foods (functional foods), fortifying yogurt with various phenolic‐rich sources, including nuts, seeds, and plants, has gained popularity (Shirani et al. 2022). Hazelnuts are a rich source of essential phytochemicals, including carbohydrates, dietary fiber, and active plant compounds, making them valuable food for human nutrition and health. They are particularly abundant in bioactive compounds such as tocopherols (vitamin E), phytosterols, and phenolic compounds, which have been shown to offer numerous health benefits. Additionally, phenolic compounds in hazelnuts exhibit anti‐inflammatory, antioxidant, and anticancer properties, contributing to their functional potential in a balanced diet (Aysu et al. 2022; Maleki et al. 2015; Oliveira et al. 2008). Fortifying dairy products with natural compounds is a well‐known method for delivering beneficial prebiotics, probiotics, and bioactive substances. Additionally, incorporating natural ingredients into yogurt not only boosts the levels of bioactive substances but also enhances the release of bioactive peptides, which contribute to improved antioxidant activity compared to regular yogurt. The FDA defines functional foods as foods or food components that offer health benefits beyond their basic nutrient content, providing biologically active compounds that can improve well‐being when consumed in adequate amounts (Shirani et al. 2022). This study aimed to increase the added value of hazelnuts, the most commercially important crop cultivated in Giresun Province, by producing hazelnut milk and incorporating it into cow's milk at varying ratios to develop probiotic yogurt with enhanced functional properties. The resulting yogurt samples were evaluated in terms of their physicochemical, microbiological, bioactive, and textural characteristics.

## 2 Materials and Methods

### 2.1 Material

In this study, the Giresun Tombul hazelnut ( *Corylus avellana* L.) variety was used. Raw hazelnut kernels were purchased from the Giresun Integrated Hazelnut Processing Facility (EFİT; Fiskobirlik). The probiotic yogurt cultures employed in this research (lyophilized commercial cultures) were obtained from Doğadan Bizim Food and Dairy Products Industry and Trade Ltd. Co. (Eyüp, Istanbul). The probiotic yogurt cultures consist of *Lactobacillus delbrueckii* ssp. *bulgaricus*, *Streptococcus thermophilus* , *Lactobacillus acidophilus* , *Lacticaseibacillus rhamnosus*, *Lactiplantibacillus plantarum*, and *Bifidobacterium animalis* ssp. *lactis* strains. All chemicals used were of analytical grade and were purchased from Merck Chemical Co. (Darmstadt, Germany) and Sigma‐Aldrich Co. (St. Louis, MO, USA).

### 2.2 Production of Hazelnut Milk‐Added Probiotic Yogurts

For obtaining hazelnut milk, the acquired hazelnuts were ground for 10 min using a blender (Waring laboratory blender, Conair Corporation, Stamford, CT, USA). The blended hazelnuts (200 g) were homogenized with 1 L of water for 1 h using an ultrasonic homogenizer (sonicator; BANDELIN SONOPULS HD 3100, Landsberg, Berlin, Germany). The obtained hazelnut milk was added to pasteurized cow's milk at proportions of 10%, 20%, 30%, 40%, and 50% (CH‐1, CH‐2, CH‐3, CH‐4 and CH‐5, respectively) according to the ratios determined in preliminary trials. The mixture was transferred into sterile glass jars and pasteurized at 70°C for 15 min using a water bath. After pasteurization, the hazelnut milk was cooled to 43°C, and a probiotic yogurt culture was added at a 4% ratio. The bacterial population at the time of inoculation was approximately 10^8^ CFU/mL, as provided by the commercial starter culture specification. Fermentation continued until the pH reached 4.7 at 41°C. After incubation, the yogurt samples were cooled and held at 4°C for 24 h to allow initial textural and structural maturation. This period, commonly referred to as post‐acidification or setting, enables the gel network to stabilize. Subsequently, the samples were stored at 4°C for an additional 21 days for further evaluation of quality parameters during refrigerated shelf‐life. As the control group for the analysis, probiotic yogurt was prepared using only cow milk and a 4% (w/v) ratio of lyophilized commercial probiotic yogurt culture, without the addition of hazelnut milk. The fermented probiotic hazelnut milks were subjected to physicochemical, microbial, bioactivity, and textural analyses on days 1, 7, 14, and 21 of storage to determine quality parameters.

### 2.3 Physicochemical Analyses

Throughout the storage period, the analysis of samples included the determination of dry matter, ash, protein, titratable acidity, pH, and serum separation (mL) following the protocol outlined by Kalkan et al. (2018). The water activity of the samples was gauged using a water activity meter (Aqualab 4TE, METER Group Inc. USA). To obtain water activity values, samples were positioned in the dedicated compartment of the standardized device at room temperature. The color parameters of yogurt samples, specifically L (lightness), a* (red‐green), and b* (yellow‐blue), were measured using a CR‐410 colorimeter (Konica Minolta, Tokyo, Japan) on a standard white calibration plate. Measurements were taken by gently placing the probe directly on the surface of the yogurt without applying pressure, using transparent polypropylene containers (diameter: 9 cm, depth: 1 cm) filled to a uniform level to avoid background interference. The total color difference (ΔE) was calculated using the standard CIE formula: ΔE = √[(L* – *L*_0_*)^2^ + (a* – *a*_0_*)^2^ + (b* – *b*_0_*)^2^], where *L*_0_*, *a*_0_*, and *b*_0_* represent the color values of the control sample.

### 2.4 SEM (Scanning Electron Microscope) Analysis

After the preparation and refrigerated maturation of yogurt samples, lyophilization was performed on the 1st day of storage using FreeZone 2.5 (Labconco, Kansas, MO, USA) at −50°C and an average pressure of 0.45–1 mbar for pore analysis with a scanning electron microscope (SEM; SU1510; Hitachi, Tokyo, Japan). Prior to SEM imaging, lyophilized samples were securely attached to sample plates using double‐sided tape to ensure adequate conductivity and enhance image quality. Additionally, a gold coating was applied to the samples utilizing a sputter coater method (SBC‐900‐C Single Target Plasma Sputter Coater). Micrographs were captured at accelerating voltages ranging from 3 to 15 kV, and magnification levels were optimized for obtaining high‐quality images.

### 2.5 Texture Analysis

Yogurt samples, each contained in screw‐capped 60 mL containers, underwent texture analysis. The analysis employed a texture analyzer (TA‐XT2, Stable Micro Systems, Surrey, UK) immediately after production, on the first day of storage. For this, a 35 mm disc back extrusion fixture (A/BE‐d35) and a 5 kg load cell were used. Test parameters were as follows: pre‐test speed: 1 mm/s, test speed: 1 mm/s, post‐test speed: 10 mm/s, distance: 15 mm, and trigger force: 10 g. Four specific parameters were measured: hardness and internal stickiness were determined by the maximum positive and negative forces applied, while consistency and viscosity index were calculated from the areas under the positive and negative sides of the graph, respectively. All values presented are the average of three measurements along with their standard deviation (Balpetek Külcü et al. 2021).

### 2.6 Microbiological Analyses

Microbiological changes that may occur during storage were investigated. Total mesophilic aerobic bacteria (TMAB) and total yeast‐mold counts were determined using Plate Count Agar and Potato Dextrose Agar, following the method described by Harrigan. Enumeration of lactic acid bacteria (LAB) was carried out using De Man, Rogosa, and Sharpe (MRS) agar (Merck, Darmstadt, Germany), following the procedure outlined by Vinderola and Reinheimer (Kalkan et al. 2018). For the determination of *Streptococcus thermophilus* count in yogurt samples, M17 Agar according to TERZAGHI (M17 Agar, Merck, Germany) was employed. Incubation periods were 18–24 h at 37°C for TMAB, 48–72 h at 35°C–37°C for *Streptococcus thermophilus*, 5–7 days at room temperature for yeast and mold, and 3 days at 35°C–37°C in an anaerobic jar for lactic acid bacteria. Enumeration of probiotic bacteria was carried out using de Man, Rogosa, and Sharpe (MRS) agar for *Lactobacillus delbrueckii* ssp. *bulgaricus*, *Streptococcus thermophilus* , *Lactobacillus acidophilus* , *Lacticaseibacillus rhamnosus*, *Lactiplantibacillus plantarum*, and was carried out using MRS agar supplemented with 0.05% L‐cysteine for the *Bifidobacterium* spp. All plates were incubated anaerobically at 37°C for 72 h.

### 2.7 Total Phenolic Content Analysis

The total phenolic content of the yogurt samples was measured using the Folin–Ciocalteu colorimetric method, following a modification of the procedure outlined by Maleki et al. (2015). Prior to analysis, phenolic compounds were extracted from yogurt samples using an ethanol‐based extraction protocol. Briefly, 10 g of each yogurt sample was mixed with 20 mL of 80% ethanol and homogenized using a vortex mixer. The mixture was then centrifuged at 5000 rpm for 15 min at 4°C. The resulting supernatant was collected and used for spectrophotometric measurement. A 1.0 mL aliquot of the extract was mixed with 0.5 mL of 1 N Folin–Ciocalteu reagent and 6.0 mL of distilled water using a vortex mixer (Heidolph Reax Top vortex, Heidolph Instruments, Schwabach, Germany) and shaken for 1 min. After the initial mixing, 1.0 mL of 5% sodium carbonate solution was added, and the mixture was shaken again. Following incubation for 60 min at room temperature in the dark, absorbance was measured at 725 nm using a UV–visible spectrophotometer (Hach DR6000, Lange GmbH, Germany), with distilled water as the blank. Total phenolic content was calculated using a calibration curve prepared with gallic acid (concentration range: 1–1500 μg/mL), and results were expressed as milligrams of gallic acid equivalents (GAE) per milliliter of sample.

### 2.8 Determination of DPPH Radical Scavenging Activity

The DPPH radical possesses a lone unpaired electron, and its absorbance undergoes changes upon receiving a radical electron or hydrogen radical. To assess the free radical scavenging potential of yogurt samples, the methodology outlined by Maleki et al. (2015) was adopted. In this procedure, 1.0 mL of the sample was combined with 3.0 mL of DPPH methanol solution in tubes. After 30 min of incubation in darkness at room temperature, the mixture underwent agitation using a vortex mixer (Heidolph Reax Top vortex, Heidolph Instruments, Schwabach, Germany). Subsequently, the absorbance of the samples at 517 nm was measured against a blank sample (methanol) employing a UV–visible spectrophotometer (Hach DR6000, Lange GmbH, 189, Germany). The percentage inhibition of the DPPH radical was then calculated using the following equation (Equation 1).

(1) $$\text{Inhibition}\;\left(\%\right)=\left[1-\left(\text{Asample}/\text{Acontrol}\right)\right]\times 100$$

In the equation, Acontrol represents the absorbance value of a control reaction (containing all reagents except the test compound), while Asample is the absorbance value of the test compound.

### 2.9 Statistical Analyses

Statistical analyses were conducted utilizing the statistical software package “SPSS 20.0 for Windows” (SPSS Inc., Chicago, IL, USA). To assess the significance of variations between tests, a one‐way analysis of variance (ANOVA) was employed. Additionally, for comparing differences between groups, the Duncan's multiple comparison test was applied as an alternative approach (*p* ≤ 0.05).

## 3 Results and Discussion

### 3.1 Physicochemical Properties of Yogurt Samples

**FIGURE 1:**
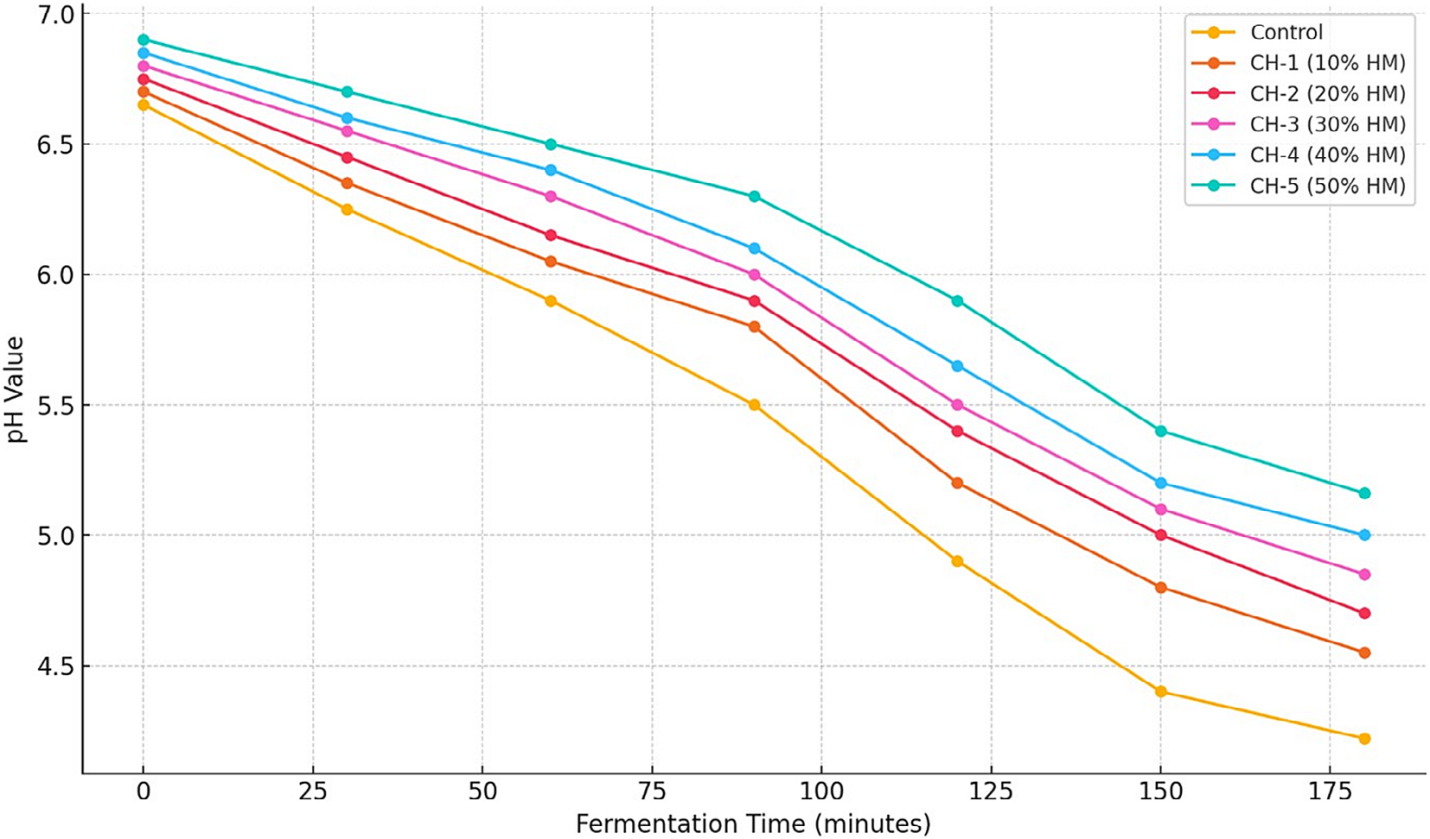
pH alteration graph for each yogurt sample during fermentation.

The pH values of probiotic yogurt samples during fermentation showed a gradual decrease over time for all formulations, as expected due to lactic acid production by starters and probiotic bacteria. However, the rate of acidification differed based on the hazelnut milk ratio. The control group (100% cow milk) exhibited the fastest pH reduction, reaching 4.22 after 180 min. In contrast, samples containing higher ratios of hazelnut milk, especially CH‐5 (50% hazelnut milk), demonstrated a slower acidification rate, ending at a higher final pH of 5.16. This trend may be attributed to the lower buffering capacity and reduced fermentable lactose content in hazelnut milk compared to cow milk, which affects microbial metabolism and lactic acid synthesis. Similar findings were reported by Yilmaz‐Ersan and Topcuoglu (2022), who observed delayed acidification in almond milk‐based probiotic yogurt systems. Slower acidification may also influence the gelation time, texture development, and microbial stability of the final product. The pH alteration graph for each yogurt sample during fermentation was shown at Figure 1.

The proximate composition of the hazelnut milk used in this study was determined as follows: protein 2.00% ± 0.71%, fat 1.20% ± 0.15%, ash 0.48% ± 0.07%, and dry matter 15.22% ± 0.68%. These compositional values were taken into account when interpreting the physicochemical and textural changes observed in the yogurt samples. The physicochemical properties of yogurt produced with nut‐based plant milks are critical determinants of its overall quality, safety, nutritional value, and consumer acceptability (Shirani et al. 2022) As seen in Table 1, the physicochemical properties of the yogurt samples varied within the following ranges: dry matter content, 12.22% ± 0.65% to 16.43% ± 0.82%; ash content, 0.43% ± 0.10% to 0.81% ± 0.00%; protein content, 3.35% ± 0.35% to 4.60% ± 1.63%; pH, 4.22 ± 0.00 to 5.16 ± 0.00; lactic acid content (LA%), 0.57 ± 0.11 to 0.74 ± 0.03; serum separation (mL/25 g), 5.00 ± 0.00 to 8.00 ± 0.70; and water activity, 0.9834 ± 0.00 to 0.9985 ± 0.00.

**TABLE 1:** Physicochemical properties of samples.

Storage days	Samples	Dry matter* (%)	Protein* (%)	Ash* (%)	pH value*	Water activity*	Serum separation* (mL/25 g)	LA* (%)
1	Control	12.29 ± 0.52^aA^	3.35 ± 0.35^aA^	0.80 ± 0.01^cA^	4.27 ± 0.00^aA^	0.9985 ± 0.00^cB^	8.00 ± 1.41^cC^	0.74 ± 0.03^bA^
	CH‐1	12.22 ± 0.65^aA^	3.89 ± 0.92^bA^	0.77 ± 0.01^cA^	4.25 ± 0.05^aA^	0.9865 ± 0.00^bA^	7.00 ± 1.41^bAB^	0.73 ± 0.01^bA^
	CH‐2	12.65 ± 0.02^aA^	3.89 ± 1.63^bA^	0.58 ± 0.04^bA^	4.74 ± 0.00^abB^	0.9859 ± 0.00^bA^	6.00 ± 0.00^abA^	0.72 ± 0.00^bA^
	CH‐3	12.70 ± 0.23^aA^	3.93 ± 1.56^bA^	0.65 ± 0.03^abA^	4.84 ± 0.00^abB^	0.9853 ± 0.00^bA^	5.85 ± 1.76^aA^	0.71 ± 0.00^bA^
	CH‐4	13.50 ± 0.21^bA^	4.06 ± 1.48^bcA^	0.63 ± 0.01^abA^	5.00 ± 0.00^bB^	0.9854 ± 0.00^bAB^	6.00 ± 1,41^abA^	0.63 ± 0.02^abA^
	CH‐5	15.22 ± 0.68^cA^	4.11 ± 1.98^cA^	0.48 ± 0.10^aA^	5.16 ± 0.00^bB^	0.9831 ± 0.00^aA^	5.50 ± 0.70^aA^	0.58 ± 0.12^aA^
7	Control	13.01 ± 0.19^bB^	3.44 ± 0.92^aA^	0.76 ± 0.01^bA^	4.22 ± 0.00^aA^	0.9855 ± 0.00^aA^	6.25 ± 1.06^bA^	0.73 ± 0.01^bA^
	CH‐1	12.44 ± 0.32^aA^	3.93 ± 1.27^bA^	0.72 ± 0.00^bA^	4.27 ± 0.00^aA^	0.9868 ± 0.00^abA^	6.75 ± 0.35^bAB^	0.72 ± 0.01^bA^
	CH‐2	13.04 ± 0.15^bB^	4.11 ± 1.27^bB^	0.75 ± 0.02^bB^	4.55 ± 0.02^aAB^	0.9850 ± 0.00^aA^	5.75 ± 0.35^aA^	0.69 ± 0.03^bA^
	CH‐3	12.32 ± 0.17^aA^	4.06 ± 1.91^bAB^	0.71 ± 0.00^bA^	4.52 ± 0.00^aAB^	0.9861 ± 0.00^abA^	6.12 ± 0.53^bAB^	0.69 ± 0.01^bA^
	CH‐4	14.89 ± 0.14^cB^	4.15 ± 1.63^bA^	0.66 ± 0.00^bA^	4.87 ± 0.00^abB^	0.9867 ± 0.00^abB^	5.75 ± 0.35^aA^	0.64 ± 0.02^abA^
	CH‐5	15.56 ± 1,26^cA^	4.24 ± 1.77^bcAB^	0.43 ± 0.08^cA^	5.00 ± 0.00^bB^	0.9847 ± 0.00^aAB^	5.50 ± 0.70^aA^	0.57 ± 0.11^aA^
14	Control	12.86 ± 0.89^aA^	3.57 ± 0.71^aA^	0.81 ± 0.00^bA^	4.22 ± 0.00^aA^	0.9859 ± 0.00^abA^	7.25 ± 1.06^bcB^	0.72 ± 0.03^bA^
	CH‐1	13.22 ± 0.22^abB^	3.93 ± 1.27^abA^	0.77 ± 0.02^bA^	4.25 ± 0.00^aA^	0.9867 ± 0.00^bA^	6.50 ± 0.70^bA^	0.73 ± 0.01^bA^
	CH‐2	13.08 ± 0.40^aB^	4.33 ± 1.20^bB^	0.80 ± 0.02^bB^	4.38 ± 0.00^aA^	0.9858 ± 0.00^abA^	6.00 ± 0.00^abA^	0.69 ± 0.00^bA^
	CH‐3	13.43 ± 0.64^bB^	4.42 ± 1.34^bB^	0.73 ± 0.01^bA^	4.47 ± 0.00^aAB^	0.9867 ± 0.00^bAB^	5.75 ± 1.06^aA^	0.68 ± 0.01^bAB^
	CH‐4	14.53 ± 0.14^cB^	4.47 ± 1.41^bB^	0.73 ± 0.00^bA^	4.38 ± 0.14^aA^	0.9844 ± 0.00^aA^	5.25 ± 0.35^aA^	0.64 ± 0.01^abA^
	CH‐5	15.03 ± 0.58^cA^	4.56 ± 1.56^bB^	0.63 ± 0.02^aB^	4.44 ± 0.00^aA^	0.9845 ± 0.00^aAB^	5.00 ± 0.00^aA^	0.57 ± 0.11^aA^
21	Control	13.07 ± 0.00^bB^	3.57 ± 0.71^aA^	0.80 ± 0.00^bA^	4.27 ± 0.00^aA^	0.9878 ± 0.00^bB^	8.00 ± 0.70^cC^	0.72 ± 0.03^bA^
	CH‐1	12.23 ± 0.27^aA^	4.24 ± 1.06^bB^	0.76 ± 0.00^bA^	4.24 ± 0.00^aA^	0.9862 ± 0.00^bA^	6.62 ± 0.88^bA^	0.72 ± 0.03^bA^
	CH‐2	13.27 ± 0.29^aB^	4.24 ± 1.06^bB^	0.76 ± 0.00^bB^	4.29 ± 0.00^aA^	0.9869 ± 0.00^bAB^	6.25 ± 0.35^bAB^	0.69 ± 0.00^bA^
	CH‐3	12.73 ± 0.03^aA^	4.33 ± 1.20^bB^	0.68 ± 0.01^aA^	4.29 ± 0.00^aA^	0.9869 ± 0.00^bAB^	5.50 ± 0.70^aA^	0.67 ± 0.00^bAB^
	CH‐4	14.46 ± 1.76^cB^	4.47 ± 0.71^bB^	0.67 ± 0.01^aA^	4.33 ± 0.00^aA^	0.9869 ± 0.00^bB^	5.50 ± 0.70^aA^	0.63 ± 0.01^abA^
	CH‐5	16.43 ± 0,82^dB^	4.60 ± 1.63^bB^	0.64 ± 0.01^aB^	4.34 ± 0.61^aA^	0.9834 ± 0.00^aA^	5.50 ± 0.70^aA^	0.58 ± 0.12^aA^

*Note:* a–c: Different superscript letters within the same column mean significant differences between the same storage days × different samples (*p* < 0.05); A, B: Different superscript letters within the same column mean significant differences between the different storage days × same samples.

* Mean ± standard deviation (*n* = 3).

Milk composition significantly influences the dry matter content of yogurt, affecting its texture, flavor, and nutritional profile (Topcuoglu 2019). The dry matter values of the samples increased according to the ratio of added hazelnut milk (*p* ≤ 0.05). The lowest dry matter values were observed in probiotic yogurt samples with 10% hazelnut milk on the first day of storage, while the highest dry matter values were observed in probiotic yogurt samples with 50% hazelnut milk on the last day of storage, which was 21 days (*p* ≤ 0.05). Similarly, previous studies have reported that the incorporation of plant‐based milk into cow's milk leads to an increase in the dry matter content of yogurt samples (Hassan et al. 2012; Shrestha and Yadav 2018). The dry matter values of probiotic fermented beverages produced with sesame and pumpkin seed milk ranged from 9.92% to 14.17% (Hassan et al. 2012). In another study conducted by Shrestha and Yadav (2018) aimed at evaluating the effect of corn milk addition (0%, 10%, 20%, 25%, and 30%) on the quality of soy yogurt, the dry matter content of the samples was determined to be 22.03% ± 0.31.

The highest ash values were observed in the control group probiotic yogurt samples produced only from cow's milk, while the lowest ash values were found in samples containing 50% hazelnut milk (*p* ≤ 0.05). Ash content in yogurt is commonly associated with its overall mineral composition including sodium, calcium, potassium, and magnesium. Although hazelnut milk contains minerals, its overall mineral concentration is generally lower compared to cow's milk, which could explain the reduction in total ash values. Additionally, it is generally well established that the ash content of yogurt is closely associated with its sodium chloride (NaCl) concentration. Therefore, the reduction in NaCl content resulting from the incorporation of hazelnut milk into cow's milk may contribute to the observed decrease in the ash content of the yogurt samples (Somer 2013). Consistent with the findings of the present study, Isanga and Zhang (2009) reported that yogurt samples produced using peanut milk exhibited a total ash content of 0.61% ± 0.02%.

The protein content of hazelnut milk is higher compared to other plant‐based milks. Additionally, fermentation and the interactions between protein and other ingredients can also influence protein retention and structure in the final yogurt product (Oliveira et al. 2008; Aysu et al. 2022). In accordance with previous studies, the protein values increased with the increasing ratio of hazelnut milk (*p* ≤ 0.05). The lowest protein values were observed in the control group probiotic yogurt samples produced only from cow's milk, while the highest protein values were observed in probiotic yogurt samples with 50% hazelnut milk (*p* ≤ 0.05). During the storage period, it was observed that the protein content of the probiotic yogurt samples increased in parallel with the proportion of hazelnut milk added (*p* ≤ 0.05). Although hazelnut milk used in this study had a lower crude protein content (2.00% ± 0.71%) compared to cow milk (3.05% ± 0.08%), the total protein values in yogurt increased from 3.35% in the control group to 4.11% in CH‐5. This unexpected trend may be explained by multiple factors. First, the addition of hazelnut milk contributed to a significant increase in dry matter content (from 12.22% to 15.22%), potentially concentrating the protein fraction in the final product. Second, the protein–polyphenol interactions arising from hazelnut components might have influenced protein precipitation behavior during fermentation and affected quantification. Additionally, the presence of suspended particulate matter from hazelnut residues could have contributed to the total nitrogen content, thus increasing the measured protein levels using the Kjeldahl method. Similar increases in protein content with plant‐based milk additions have also been reported by Isanga and Zhang (2009) in peanut milk yogurt (5.17%) and Biswas (2013) in coconut milk‐based yogurt (3.16%–3.72%). While these findings vary due to formulation and processing differences, they are consistent with the literature trend.

According to the pH values presented in Table 1, the initial pH of yogurt samples increased significantly with the addition of hazelnut milk (*p* ≤ 0.05), ranging from 4.22 in the control to 5.16 in CH‐5. However, during the 21‐day storage period, a gradual decrease in pH was observed specifically in the CH‐2, CH‐3, CH‐4, and CH‐5 samples, while the pH of the control (CH‐0) and CH‐1 remained relatively stable. This slight pH reduction in higher hazelnut milk ratios may be attributed to ongoing microbial metabolism—particularly by probiotic strains such as *Lactobacillus acidophilus* and *Bifidobacterium animalis* —which can continue limited fermentation and acid production under refrigeration. In contrast, lower hazelnut milk ratios may have resulted in more complete acidification during fermentation, leaving less residual substrate for further microbial activity during storage. Moreover, the buffering capacity of hazelnut milk is lower than that of cow milk, which could result in more pronounced pH fluctuations under continued metabolic activity. Similar post‐fermentation pH trends have been reported in plant‐based yogurt systems by Arslan (2018) and Shori (2013), where ongoing metabolic reactions of probiotic strains caused slight acidification during storage. Previous studies on the fermentation and storage of plant‐based milk products have reported variations in pH values over time. For example, Arslan (2018) observed that yogurt samples produced from pistachio milk exhibited a continuous decline in pH during the first 14 days of storage, decreasing from 4.69 to 4.36, followed by a slight increase to 4.40 on the 21st day. Differently, Bernata et al. (2015), in their study on probiotic yogurt produced from almond milk using *Limosilactobacillus reuteri* and *Streptococcus thermophilus* , reported stable pH values ranging from 4.63 to 4.65 after 28 days of storage. The stable pH values (4.63–4.65) reported by Bernata et al. (2015) are likely due to the limited fermentable sugars in almond milk, the buffering capacity of the plant matrix, and the reduced metabolic activity of the probiotic strains during cold storage. Another study by Hassan et al. (2012) reported that the pH values of probiotic fermented beverages produced with sesame and pumpkin seed milk ranged from 4.34 to 7.83. In a study conducted by Shrestha and Yadav (2018) aimed at evaluating the effect of corn milk addition (0%, 10%, 20%, 25%, and 30%) on the quality of soy yogurt, the pH values of the samples were determined to be 4.58 ± 0.03.

The titratable acidity (LA%) values of the samples were found to decrease with the increasing ratio of hazelnut milk added to cow's milk (*p* ≤ 0.05). According to the Turkish Food Codex Fermented Dairy Products Regulation (Ozturkoglu‐Budak et al. 2016), the titratable acidity of yogurt should range between 0.6% and 1.5% lactic acid (LA%). The results obtained in the current study showed that most yogurt samples were within this specified range throughout storage. However, the CH‐5 sample, which contained 50% hazelnut milk, exhibited slightly lower acidity values during the entire storage period (minimum 0.58%), falling just below the lower limit. This slight deviation may be attributed to the lower fermentable carbohydrate content and buffering capacity of hazelnut milk compared to cow milk, which can limit acid development by lactic acid bacteria. Despite this, CH‐5 maintained adequate microbiological viability and showed no signs of spoilage, indicating acceptable quality from a safety and probiotic standpoint. Similarly, Bernata et al. (2015) reported that the acidity of samples resulting from the fermentation of hazelnut milk was lower, which was explained by the lower buffering capacity of hazelnut milk compared to cow's milk. Ozturkoglu‐Budak et al. (2016) found that yogurts containing nuts had lower acidity depending on the amount of dietary fiber and protein content in the nuts. These findings are in agreement with the results of the current study, particularly the CH‐5 sample, which showed the lowest titratable acidity values (below 0.6% LA) during storage. The reduced acidification observed in hazelnut milk‐enriched yogurts can be attributed to both the lower fermentable sugar content and buffering capacity of hazelnut milk, as well as the presence of dietary fiber and plant proteins, which may inhibit acid development by interfering with bacterial metabolism or altering the gel matrix. Raikos et al. (2020) stated that the titratable acidity values of oat milk‐based yogurts ranged from 0.19% to 0.50%. In a different study by Shori et al. (2022), the LA (%) values of yogurt samples obtained with the addition of *L. rhamnosus* , *L. casei* , and *L. plantarum* cultures to cashew milk were determined to be 0.54% ± 0.01. Mattison et al. (2020) reported that the pH and LA of commercial cashew milk‐based yogurt were 4.46% and 0.53%, respectively. According to the analysis results shown in Table 1, the serum separation values decreased significantly with the increasing proportion of hazelnut milk added to cow's milk (*p* ≤ 0.05). the reduction in syneresis may be attributed to the increased total solids in the yogurt matrix, particularly contributed by the dietary fiber and plant‐based components present in hazelnut milk. These elements are known to enhance gel formation and network density, thereby contributing to better water retention. Although a slight decrease in ash content was observed with increasing hazelnut milk ratio—possibly due to its lower mineral content compared to cow's milk—this trend does not appear to be directly related to syneresis in this study. In fact, the improved microstructure and increased total solids content (especially dry matter) are more plausible contributors to the reduction in serum separation. This is supported by SEM analysis and the increased values of textural parameters, indicating a more compact gel structure. Similar findings have been reported in studies using nut‐based milk alternatives, which suggest improved water‐holding capacity due to their functional composition rather than mineral content alone. Additionally, factors such as low protein, fat, and mineral content in the milk used, improper curd handling, and low acid development during production can affect serum separation (Mukisa and Kyoshabire 2010). It is believed that the addition of hazelnut milk positively improved the protein, fat, and mineral content in the product and contributed to lower serum separation values compared to other studies. Indeed, the increase in the ratio of hazelnut milk added to cow's milk was associated with an increase in the dry matter content of the probiotic yogurt samples, supporting this result. Water activity is crucial in food technology as it directly affects the physical, chemical, and microbiological stability of foods, thereby influencing food quality (Aktaş and Gölge 2022). As shown in Table 1, the water activity values exhibited a trend that paralleled the dry matter content of the samples. The probiotic yogurt samples with the highest dry matter had the lowest water activity values, while those with lower dry matter had the highest water activity values.

Table 2 displays the color values of the samples. As observed in Table 2, it was determined that the color values of the samples varied significantly depending on the proportion of hazelnut milk added to cow's milk (*p* ≤ 0.05). The L* values, which indicate lightness, showed a decreasing trend in CH‐1, CH‐2, and CH‐3 on the first day, suggesting a darker appearance compared to the control. However, in CH‐4 and CH‐5, L* values increased, resulting in a lighter color than the control—particularly in CH‐5, where the L* value was even higher than the control. This unexpected trend might be associated with the increased number of light‐colored components in higher concentrations of hazelnut milk, which could influence the light scattering properties of the product matrix. Regarding the a* values (red‐green coordinate), an increase was observed with the increasing proportion of hazelnut milk, indicating a shift towards the red spectrum. This suggests that hazelnut milk may contribute pigments that enhance the reddish tones in the samples. Similarly, b* values (yellow‐blue coordinate) increased, indicating a higher yellow intensity with more hazelnut milk. These changes are likely related to the presence of carotenoids, flavonoids, and other phenolic pigments in hazelnut milk, which contribute to the color profile of the yogurt samples. Whiteness in milk is due to the presence of colloidal particles such as milk fat globules and casein micelles, which can scatter light in the visible spectrum. The observed differences in yogurt samples indicate an interaction between the protein source and pigments in milk types. Particularly, carotenoids play a significant role in shaping the color profile of milk and, consequently, yogurt. The fluctuations in color parameters observed in the samples can be elucidated by the impact of pigments like carotenoids and flavonoids, which are accountable for the red, yellow, and orange hues in milk products, including enriched plant‐based milks (Yılmaz Ersan et al. 2017; Chudy et al. 2020). Similar to our study, Mattison et al. (2020) reported an average L* (lightness/darkness) value of 84.67 ± 0.47 for cashew nut‐based yogurts. The average a* (red/green) value of the same samples was 0.73 ± 0.15, indicating a very slight redness, and the average b* (yellow/blue) value was 8.89 ± 0.53, indicating a slight yellowness.

**TABLE 2:** Color properties of samples.

Samples	Storage days			
	1	7	14	21
Control				
*L* *	70.03 ± 0.66^abB^	71.36 ± 0.33^bC^	75.35 ± 0.85^bC^	72.10 ± 0.23^bB^
*a* *	−1.57 ± 0.10^aA^	−1.54 ± 0.11^aA^	−1.19 ± 0.02^aA^	−1.48 ± 0.04^aA^
*b* *	9.41 ± 0.43^aA^	9.55 ± 0.11^aA^	10.03 ± 0.08^aA^	9.66 ± 0.31^aA^
Δ*E* *	26.75 ± 0.53^bB^	25.50 ± 0.09^bA^	21.88 ± 0.77^aA^	24.83 ± 0.21^bA^
CH‐1				
*L* *	68.38 ± 0.69^aB^	69.17 ± 0.71^abB^	73.00 ± 0.32^bB^	70.38 ± 1.67^aA^
*a* *	−0.68 ± 0.04^bcB^	−0.56 ± 0.22^bB^	−0.30 ± 0.02^aB^	−0.78 ± 0.17^cB^
*b* *	10.04 ± 0.24^aB^	10.10 ± 0.22^aB^	10.55 ± 0.05^aA^	9.90 ± 0.43^aA^
Δ*E* *	28.51 ± 0.73^bC^	27.76 ± 0.73^bB^	24.25 ± 0.30^aB^	26.55 ± 1.49^bB^
CH‐2				
*L* *	68.58 ± 0.59^aB^	68.36 ± 1.90^aB^	75.45 ± 0.82^bC^	70.86 ± 0.38^aA^
*a* *	−0.08 ± 0.03^aC^	−0.59 ± 0.23^cB^	−0.16 ± 0.15^bC^	−0.73 ± 0.12^dB^
*b* *	10.69 ± 0.02^bB^	10.17 ± 0.23^bB^	11.09 ± 0.38^cB^	9.82 ± 0.17^aA^
Δ*E* *	28.50 ± 0.56^cC^	28.57 ± 1.72^cBC^	22.16 ± 0.75^aA^	26.06 ± 0.33^bB^
CH‐3				
*L* *	64.48 ± 0.80^aA^	67.42 ± 0.17^aA^	73.50 ± 0.18^bB^	71.04 ± 1.47^abAB^
*a* *	0.44 ± 0.06^aD^	0.58 ± 0.19^bC^	0.66 ± 0.04^cD^	0.45 ± 0.12^aC^
*b* *	10.40 ± 0.10^aB^	10.64 ± 0.19^aB^	11.53 ± 0.03^bB^	10.80 ± 0.52^aB^
Δ*E* *	32.37 ± 0.76^dD^	29.59 ± 0.30^cC^	24.12 ± 0.16^aB^	26.19 ± 1.24^bB^
CH‐4				
*L* *	69.24 ± 0.53^aB^	67.86 ± 0.38^aA^	69.43 ± 0.39^aA^	69.70 ± 0.69^aA^
*a* *	1.26 ± 0.03^cF^	0.88 ± 0.19^bD^	0.68 ± 0.01^aD^	0.77 ± 0.05^abD^
*b* *	12.27 ± 0.07^bC^	11.92 ± 0.19^abC^	11.58 ± 0.10^aB^	11.21 ± 0.18^aC^
Δ*E* *	28.37 ± 0.47^abC^	29.56 ± 0.13^bC^	27.96 ± 0.38^aC^	27.59 ± 0.01^aC^
CH‐5				
*L* *	74.32 ± 0.27^bC^	67.03 ± 0.21^aA^	70.06 ± 0.44^aA^	69.48 ± 0.22^aA^
*a* *	0.99 ± 0.03^aE^	1.25 ± 0.14^abE^	1.42 ± 0.08^bE^	1.10 ± 0.06^aE^
*b* *	12.51 ± 0.10^bC^	11.52 ± 0.14^aC^	12.25 ± 0.22^bC^	11.73 ± 0.14^abC^
Δ*E* *	23.77 ± 0.24^aA^	30.22 ± 0.10^cCD^	27.59 ± 0.35^bC^	27.96 ± 0.17^bC^

*Note:* a–d: Different superscript letters within the same column mean significant differences between the same storage days × different samples (*p* < 0.05); A–F: Different superscript letters within the same column mean significant differences between the different storage days × same samples.

* Mean ± standard deviation (*n* = 3).

The ΔE values calculated to evaluate the total color differences between the samples regarding color measurements are presented in Table 2. ΔE is a quantitative measure that expresses the perceptibility of the difference between two colors by the human eye. According to the literature, a ΔE value below 1 indicates that the difference is imperceptible to the human eye; values between 1 and 2 can only be noticed by sensitive observers; and values between 2 and 3.5 are perceptible to the naked eye (Chudy et al. 2020). In this context, the fact that the ΔE values of the CH‐4 and CH‐5 groups are above 3.5 compared to the control group indicates that the color differences can be easily perceived by consumers. The increasing distinctness of this difference with higher hazelnut milk ratios supports that plant‐based pigments cause significant changes in product color. Therefore, the ΔE value has been considered an important indicator not only for the numerical differences in color parameters but also for demonstrating the sensory perceptibility of these differences.

### 3.2 The Pore Structures of Yogurt Samples

**FIGURE 2:**
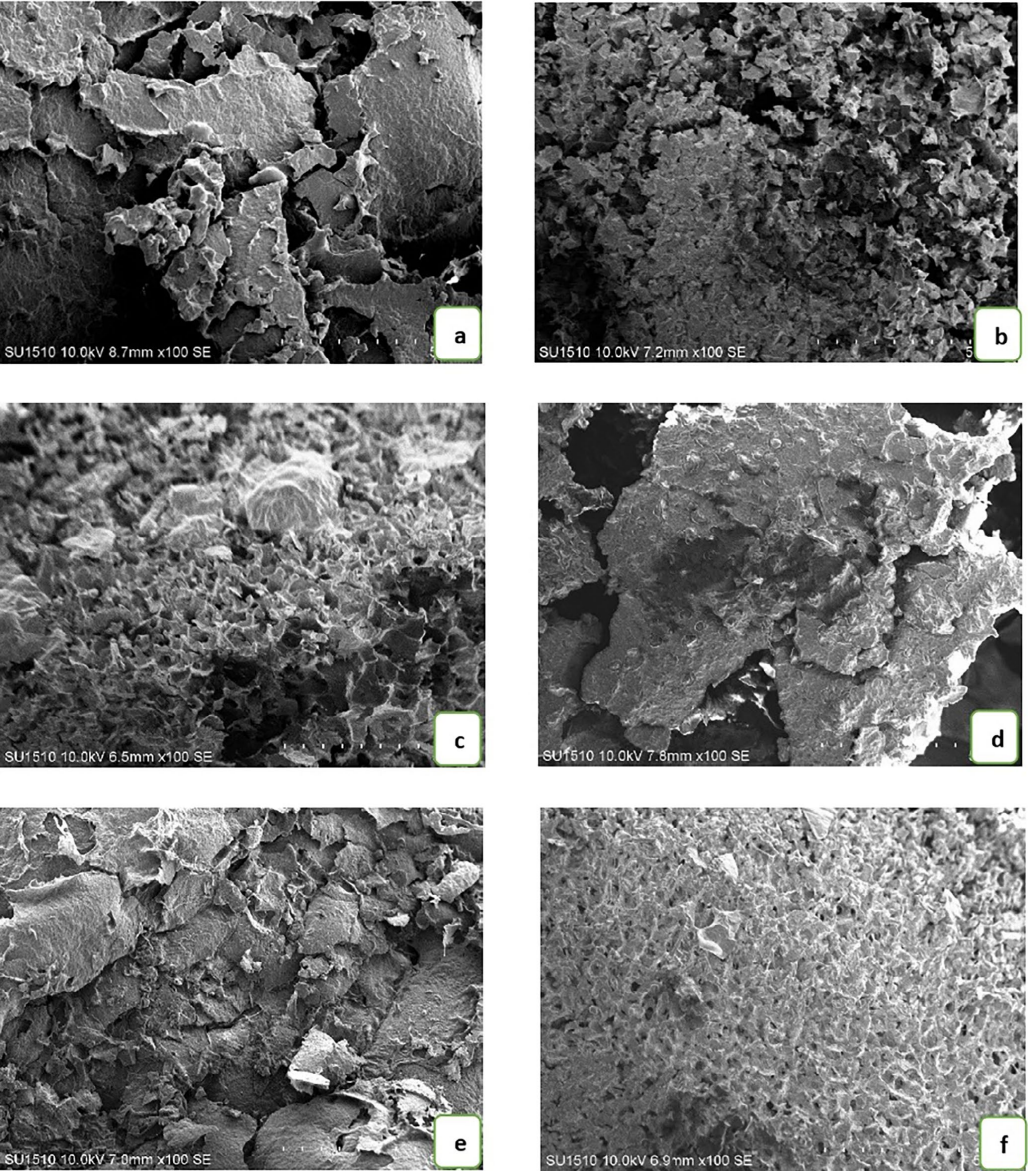
SEM images of samples (a: Control; b: CH‐1; c: CH‐2; d: CH‐3; e: CH‐4; f: CH‐5).

The pore structures of probiotic yogurt samples enriched with different ratios of hazelnut milk were examined using SEM, and the resulting micrographs are presented in Figure 2a–f. The control sample (Figure 2a), produced only from cow's milk, displayed a discontinuous, branched gel network with large, irregularly distributed voids and visible micropores, suggesting a weaker and loosely bound protein matrix. In contrast, as the concentration of hazelnut milk increased (Figure 2b–f), particularly in CH‐4 and CH‐5, the microstructure became progressively more compact, smoother, and homogeneous, with fewer and smaller voids. This denser network is likely due to the synergistic effect of hazelnut‐derived proteins and phenolic compounds interacting with milk proteins, enhancing gel compactness through increased protein–protein and protein–polyphenol interactions. The observed structural transition correlates with the increase in dry matter content and pH, and the decrease in titratable acidity and serum separation, as these physicochemical shifts contribute to improved water‐holding capacity and a more stable gel matrix. Similar findings were reported by Wang et al. (2021), where nut‐based milk proteins supported the formation of fine‐stranded gel networks. These structural improvements, observed via SEM, support the superior textural properties (e.g., higher hardness and consistency) reported for the higher hazelnut milk ratios, especially CH‐5.

### 3.3 Textural Properties of Yogurt Samples

Texture in foods is highly important not only in terms of the structure of the product but also for its sensory acceptability by consumers. In this study, the textural properties of probiotic yogurt samples produced with varying proportions of hazelnut milk were evaluated based on parameters including hardness, consistency, internal adhesiveness, and viscosity index, as shown in Table 3. The results indicated that hardness values increased from 0.43 to 1.01, and consistency values increased from 4.22 to 10.63, with higher levels of hazelnut milk addition. In contrast, internal adhesiveness (from −0.10 to −0.46) and viscosity index (from −0.03 to −0.51) decreased. The increase in hardness and consistency suggests that the yogurt structure became firmer and more cohesive, likely due to a denser gel network formed by the interaction of milk and hazelnut milk components. The decrease in internal adhesiveness implies reduced gumminess or stickiness, contributing to a smoother mouthfeel. Similarly, the reduction in viscosity index may reflect a decreased resistance to flow, indicating more fluid behavior under certain conditions. Although this may appear contradictory to the increase in consistency, these parameters measure different aspects of texture, and their divergence may be due to structural modifications affecting flow behavior during mechanical testing.

**TABLE 3:** Textural properties of samples.

Samples	Textural properties*			
	Hardness (N)	Consistency (N.s)	Inner stickiness (N)	Viscosity index (N.s)
Control	0.72 ± 0.07^c^	8.24 ± 0.85^c^	−0.25 ± 0.05^b^	−0.16 ± 0.08^b^
CH‐1	0.43 ± 0.08^a^	4.22 ± 0.45^a^	−0.10 ± 0.02^a^	−0.03 ± 0.01^a^
CH‐2	0.59 ± 0.08^b^	6.88 ± 1.13^b^	−0.21 ± 0.07^b^	−0.14 ± 0.09^b^
CH‐3	0.75 ± 0.29^c^	9.32 ± 4.19^d^	−0.36 ± 0.25^c^	−0.34 ± 0.30^c^
CH‐4	0.77 ± 0.30^c^	9.36 ± 3.64^d^	−0.45 ± 0.31^d^	−0.36 ± 0.20^c^
CH‐5	1.01 ± 0.27^d^	10.63 ± 3.16^e^	−0.46 ± 0.19^d^	−0.51 ± 0.40^d^

*Note:* a–d: Different superscript letters within the same column denote significant differences between the same storage days × different samples (*p* < 0.05).

* Mean ± standard deviation (*n* = 3).

Hardness, which represents the force required to cause the initial deformation in the mouth, is the most important parameter in the evaluation of yogurt's textural properties (Mousavi et al. 2019). The differences observed in hardness values among the samples are statistically significant (*p* ≤ 0.05). According to the analysis results, it was determined that hardness values increased with the increasing ratio of hazelnut milk added to probiotic yogurt production. The probiotic yogurt samples containing higher amounts of hazelnut milk had higher hardness, presumably due to their higher protein and total dry matter content compared to probiotic yogurt samples made only from cow's milk. This phenomenon can be explained by the higher protein content leading to an increased cross‐linking degree of the gel network, resulting in a denser and firmer gel structure (Yilmaz‐Ersan and Topcuoglu 2022). Similarly, Ozturkoglu‐Budak et al. (2016) reported that the hardness values of control group yogurt samples were higher than almond milk‐added yogurt samples. The consistency (N.s) value of products provides information about the density of the product. According to the measurement results, a high consistency value indicates a dense product with high density (Yılmaz Ersan et al. 2017; Ozcan 2013). In our study, while the addition of 10% hazelnut milk to cow's milk resulted in a decrease in consistency values compared to the control group samples, an increase in the added hazelnut milk ratio led to a significant increase in the consistency values of the produced probiotic yogurt products. Generally, the increase in fat content is associated with a softer consistency and a decrease in viscosity value in yogurt products (Joon et al. 2017). Internal stickiness, defined as the highest negative force, is analyzed as an indicator of the strength of bond formation in the analyzed product. It is also described as the degree of deformation before the product breaks or disperses in the mouth. This textural parameter is directly related to the structural integrity of yogurt, showing the strength of internal bonds, and a higher internal stickiness value is associated with a stronger gel structure in yogurt (Yılmaz Ersan et al. 2017; Ozcan 2013; Topcuoglu 2019). The differences observed in the internal stickiness values of the samples are statistically significant (*p* ≤ 0.05). In line with our study results, Arslan (2018) reported that yogurt samples produced with microfluidized hazelnut milk and milk powder added had a stronger gel structure and, consequently, higher negative internal stickiness values. On the other hand, yogurt samples produced only with hazelnut milk had lower internal stickiness values. The viscosity index of yogurt is also related to the aggregation of casein micelles and gel formation during milk fermentation. In our study, the viscosity index values increased significantly with higher hazelnut milk ratios (*p* ≤ 0.05), which can be attributed to the enhanced total solids and protein content, contributing to a denser gel matrix. Moreover, the lower serum separation values in hazelnut milk‐fortified samples suggest improved water retention capacity, which is often associated with increased viscosity. This is consistent with findings by Jiao et al. (2023), where a stronger gel network and lower syneresis led to higher viscosity in plant‐based yogurt alternatives. Therefore, the viscosity index in our samples is a direct indicator of improved textural quality due to hazelnut milk addition, which may positively influence consumer acceptance.

### 3.4 Microbiological Properties of Yogurt Samples

**FIGURE 3:**
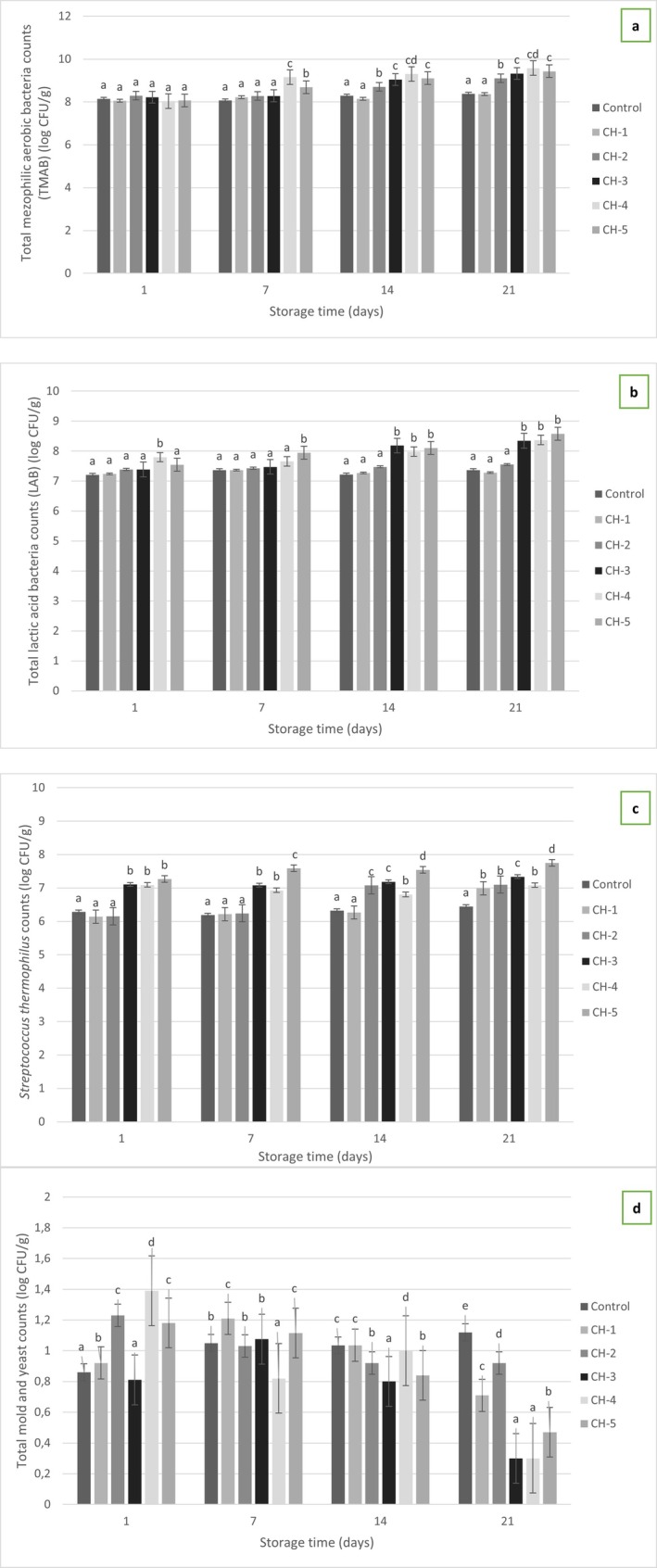
Microbial properties of samples (a: Total mesophilic aerobic bacteria counts; b: Total lactic acid bacteria counts; c: *Streptococcus thermophilus* counts; d: Total mold and yeast counts; log CFU/g).

Total Mesophilic Aerobic Bacteria (TMAB) counts of probiotic yogurt samples with varying proportions of hazelnut milk are presented in Figure 3a. Analysis indicated a statistically significant increase in TMAB counts corresponding to the higher proportions of hazelnut milk added to cow's milk (*p* ≤ 0.05). However, considering that the milk and hazelnut milk mixtures were pasteurized together, the observed increase in TMAB counts cannot be attributed directly to flavor additives or initial microbial load from hazelnut milk. Moreover, it should be noted that Plate Count Agar (PCA) primarily supports the growth of mesophilic aerobic bacteria but is not optimal for enumerating starter culture bacteria, including lactobacilli and streptococci, which typically require more selective media. Therefore, the observed TMAB values may not accurately reflect the populations of probiotic or starter culture bacteria present in the yogurt samples. Furthermore, the incubation temperature (37°C) utilized in TMAB determination may have favored conditions less optimal for precise enumeration of typical yogurt starter cultures. Consequently, the high TMAB counts reported in this study compared to existing literature (5.29–5.87 log CFU/g) might result from these methodological factors rather than an actual higher microbial load. Given these limitations, TMAB counts should be cautiously interpreted, considering multiple influencing factors, including production techniques, starter culture composition, incubation conditions, and storage parameters (Demir et al. 2023). According to the results of the analyses, it was determined that the LAB values obtained increased according to the ratio of hazelnut milk added to cow's milk (*p* ≤ 0.05; Figure 3b). During the 21‐day storage period, the total LAB counts of the yogurt samples ranged between 7.20 and 8.58 log CFU/g. These values remained well above the generally accepted threshold of 6 log CFU/g for probiotic viability, confirming that the probiotic strains remained viable throughout the storage period. This indicates that the added hazelnut milk provided a suitable environment for maintaining probiotic activity. Consistent with the findings of the present study, Yilmaz‐Ersan and Topcuoglu (2022) reported that in probiotic yogurt enriched with almond milk, the *Lactobacillus delbrueckii* subsp. *bulgaricus* counts ranged from 7.90 to 9.48 log CFU/g, while *Lactobacillus acidophilus* counts varied between 8.00 and 8.86 log CFU/g over 21 days of storage. Probiotic foods are required to contain a minimum of 6 log CFU/g viable probiotic microorganisms throughout the storage period to exert health‐promoting effects. Additionally, a daily intake of 8 to 9 log CFU/g is recommended to achieve the desired therapeutic benefits (Tamime et al. 2018). In the present study, the total lactic acid bacteria (LAB) counts observed in the yogurt samples over the 21‐day storage period consistently exceeded the recommended threshold. These findings suggest that hazelnut milk may support the viability of probiotic microorganisms, indicating its potential as a beneficial ingredient in probiotic yogurt formulations. As shown in Figure 3c, it was determined that the *S. thermophilus* values obtained increased according to the ratio of hazelnut milk added to cow milk (*p* ≤ 0.05). During the 21‐day storage period, *S. thermophilus* counts ranged between 6.14 and 7.75 log CFU/g, indicating a viable and active population of this starter culture throughout storage. The number of *Streptococcus thermophilus* increased in all yogurt samples enriched with hazelnut milk, particularly in the higher concentration groups. The initial counts on day 1 ranged between 6.14 and increased to 7.75 log CFU/g by day 21, indicating active growth and good viability of this starter culture throughout storage. This trend suggests that hazelnut milk provides a supportive matrix for *S. thermophilus* growth, likely due to its bioactive compounds and nutrient availability. Similar to our study, Yilmaz‐Ersan and Topcuoglu (2022), *S. thermophilus* counts of probiotic yogurt samples enriched with almond milk varied between 8.00–9.36 log CFU/g during the 21‐day storage period. Ozturkoglu‐Budak et al. (2016) found that the number of *S. thermophilus* increased in yogurt enriched with hazelnut, almond, pistachio, and walnut during 21 days of storage in all samples except walnut. These results are consistent with previous studies reporting that nut‐based additives can positively influence the proliferation of yogurt bacteria during storage. The literature shows that hazelnut can be an important substrate with prebiotic properties for the formation of healthy microbiota by affecting microbial fermentation in the intestine due to its high content of phenolic compounds and dietary fiber (8.1–11.54 g/100 g) (Fuso et al. 2021; Salçın and Ercoşkun 2021).

As shown in Figure 3d, it was determined that total yeast‐mold values increased significantly depending on the storage time (*p* ≤ 0.05). According to Turkish Food Codex Regulation on Fermented Milk Products, the presence of yeast and mold in yogurt should not exceed 10 CFU/g, and ideally should be absent entirely. Therefore, the detection of yeast and mold indicates secondary contamination due to improper handling, filtration, storage, and packaging after heat treatment, or insufficient attention to hygiene and sanitation rules during production and sale stages (Akarca and Tomar 2019). In our study, yeast and mold counts remained within the acceptable limits defined by the regulation throughout the storage period. Thus, it can be concluded that our production conditions met the hygiene standards effectively, and the heat treatment norms applied during production as well as the presence of probiotic microorganisms played a role in suppressing yeast and mold growth. Similarly, Wang et al. (2021) reported the absence of yeast‐mold and coliform group microorganisms in rice‐based yogurt‐like products during storage, attributing this to the inhibitory effects of probiotic strains ( *L. acidophilus* and *Bifidobacterium* spp.) on undesirable microbial flora.

### 3.5 Bioactive Properties of Yogurt Samples

**FIGURE 4:**
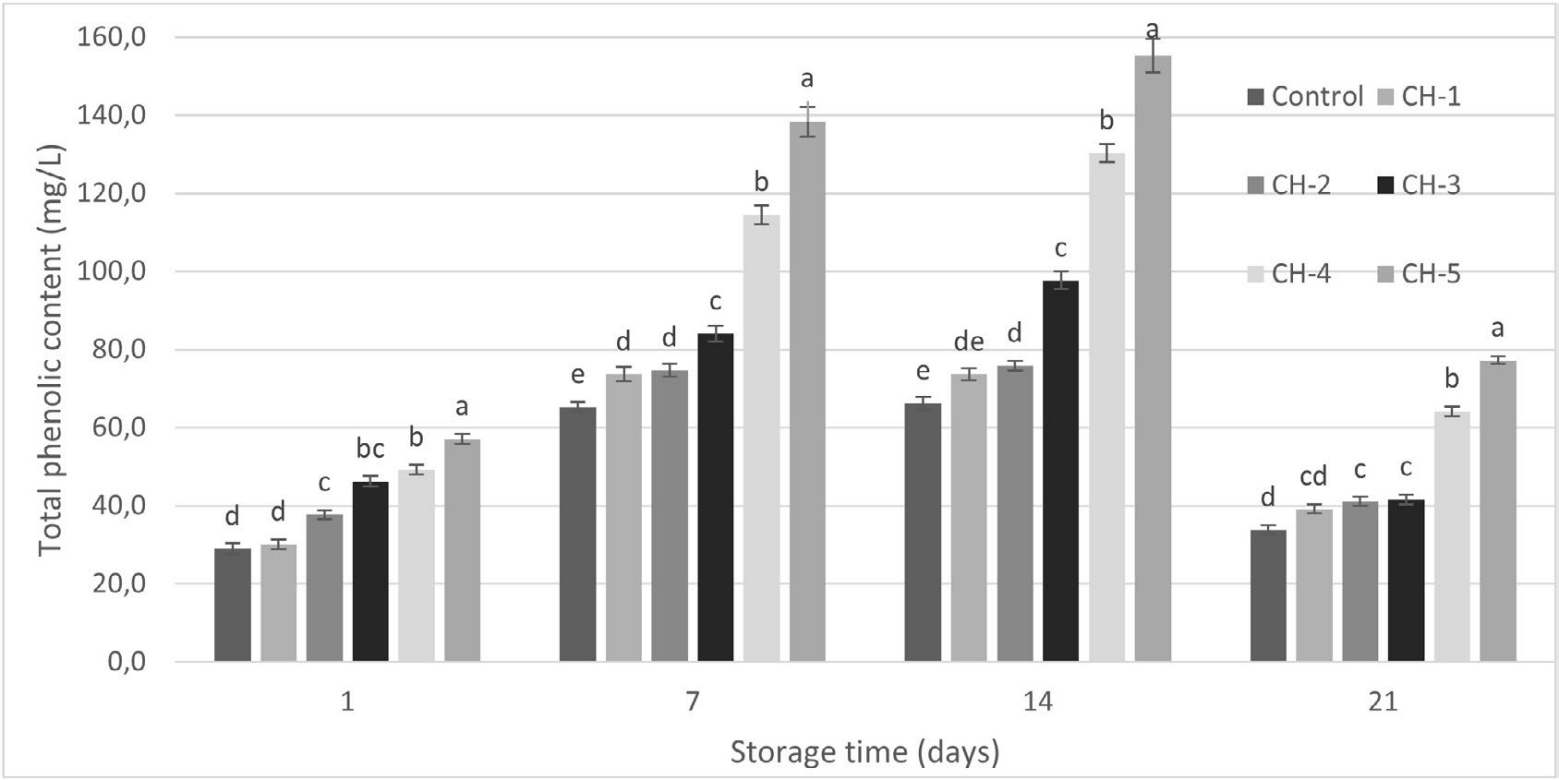
Total phenolic content of samples (mg/L).

As shown in Figure 4, the total phenolic matter (TPM) content of the yogurt samples initially increased up to the 14th day of storage, followed by a significant decrease on the 21st day, the final day of storage (*p* ≤ 0.05). Hazelnuts are well‐known for being rich in bioactive compounds, including high‐quality vegetable proteins, fiber, minerals, tocopherols, phytosterols, and phenolic compounds (Ros 2010). Among these, folic acid, selenium, n‐3 and n‐6 fatty acids, and vitamin E are particularly significant due to their reported health benefits. Dairy products, by contrast, are not typically good sources of these bioactive compounds (Ozturkoglu‐Budak et al. 2016). The present findings indicate that the TPM content of yogurt samples increased with the proportion of hazelnut milk added to cow's milk. This suggests that the bioactive compounds in hazelnut milk, including phenolics, contributed to the observed rise in TPM. Furthermore, the increase in TPM content during the cold storage period may be attributed to interactions between the added hazelnut phytochemicals and the probiotic microorganisms used during fermentation. These interactions could facilitate the release or stabilization of bioactive components, further enhancing the functional properties of the yogurt. The microbial utilization of phenolic acids, such as ferulic acid and p‐coumaric acid, during and after fermentation could potentially result in the synthesis of other acids like vanillic and p‐hydroxybenzoic acids, occurring prior to the degradation of the aromatic ring structure (Blum 1998). Additionally, the breakdown of milk proteins by microorganisms employed in yogurt production, such as the degradation of tyrosine—a phenolic side chain‐containing amino acid—may also contribute to the overall increase in the phenolic content of yogurt (Joung et al. 2016). When the literature studies on the subject are examined, the results in TPM amounts are similar. Bertolino et al. (2015) observed that phenolic matter content increased in yogurt samples prepared by adding hazelnut shell (0%, 3% and 6%) depending on the amount of hazelnut shell.

**FIGURE 5:**
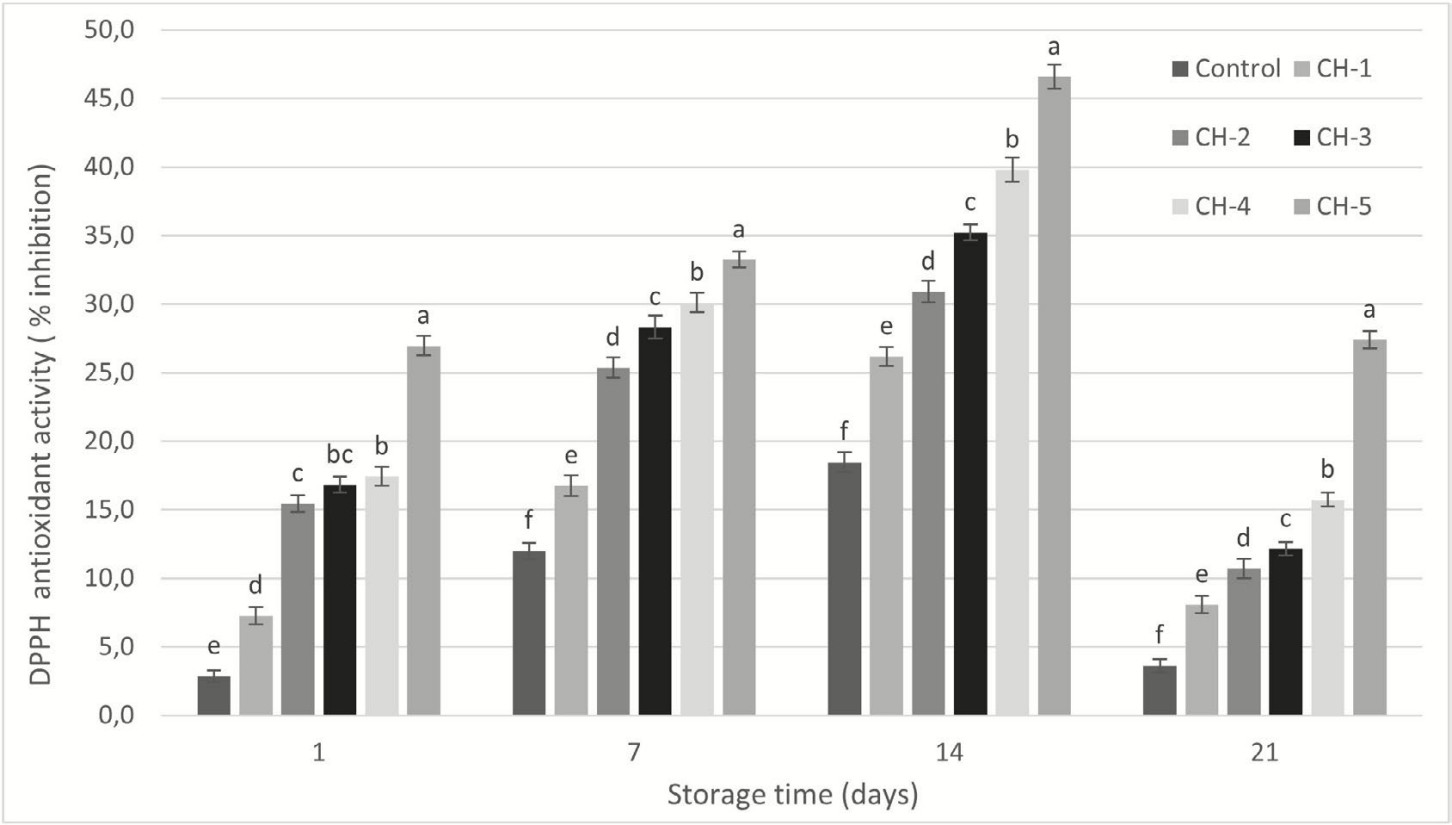
DPPH antioxidant activity (% inhibition) of samples.

It was determined that DPPH radical scavenging activity values of the samples showed an increase until the 14th day of storage similar to the TPM, and showed a significant decrease on the 21st day, the last day of storage (*p* ≤ 0.05; Figure 5). The antioxidant activity observed in probiotic products can be attributed to various metabolites produced by probiotic microorganisms, including glutathione, butyrate, and folic acid. One of the most significant antioxidant enzymes synthesized by these bacteria is superoxide dismutase (SOD), which catalyzes the conversion of superoxide radicals into water and hydrogen peroxide (Mousanejadi et al. 2023). Additionally, milk contains several endogenous enzymes—such as SOD, catalase, and glutathione peroxidase (GSH‐Px)—that inhibit radical formation or eliminate existing radicals and peroxides. These enzymes also participate in the synthesis and regeneration of non‐enzymatic antioxidants (Usta and Yılmaz‐Ersan 2013). Furthermore, milk proteins such as casein, lactoferrin, α‐lactoalbumin, and β‐lactoglobulin, as well as amino acids like tyrosine, cysteine, and tryptophan, and bioactive compounds such as oligosaccharides and peptides released during fermentation and ripening, contribute to the antioxidant potential of dairy products (Topcuoglu 2019). Collectively, these components help explain the DPPH radical scavenging activity observed in the control group samples. Hazelnut milk is a food rich in bioactive substances with antioxidant properties such as phytosterols and tocopherols (Aysu et al. 2022). As a matter of fact, Maleki et al. (2015) reported DPPH radical scavenging activities of fermented hazelnut milk as 50.47% ± 1.81%–81.65 ± 2.28%. Oliveira et al. (2008) reported that hazelnut extracts showed different antioxidant activity depending on the concentration, and the sample with a higher total phenolic content exhibited a higher antioxidant activity. When both the TPM and DPPH radical scavenging activity of the samples were evaluated during the storage period, it was observed that a significant decrease occurred on the 21st day of the storage period for both bioactive properties. This may be associated with the breakdown of phenolic substances in parallel with the increase in acidity during the storage period.

## 4 Conclusion

The findings of this study demonstrated that the incorporation of hazelnut milk at varying proportions (10%, 20%, 30%, 40%, and 50%) significantly influenced the quality characteristics of probiotic yogurt. These quality attributes were also affected by the duration of storage, with particularly notable changes observed on days 14th and 21th microstructural analysis revealed that probiotic yogurt made solely with cow's milk exhibited a discontinuous, branched protein network with prominent voids, whereas increasing the proportion of hazelnut milk resulted in a more homogeneous and smoother structure. In terms of texture, higher levels of hazelnut milk addition led to increased hardness values, with corresponding changes in consistency, internal adhesiveness, and viscosity index, all of which followed trends similar to hardness. Throughout the 21‐day storage period, all samples maintained viable probiotic counts above the minimum threshold required for health benefits. Notably, the counts of *Streptococcus thermophilus* increased with the addition of hazelnut milk. Furthermore, the total phenolic content and antioxidant activity (as indicated by lower DPPH radical scavenging values) of the yogurt samples increased proportionally with higher levels of hazelnut milk. Overall, these results suggest that hazelnut milk can be effectively used as a functional ingredient in probiotic yogurt formulations, enhancing not only the structural and textural properties but also the microbiological viability and bioactive compound content of the final product. Among all formulations, the CH‐5 sample, containing 50% hazelnut milk, exhibited the most favorable results across multiple parameters, including physicochemical properties, textural attributes, probiotic viability, and bioactive compound content. Based on these findings, CH‐5 can be considered the most optimal formulation. These results highlight the importance of selecting appropriate proportions of hazelnut milk in probiotic yogurt production to develop a functional food product that aligns with consumer preferences while offering potential health benefits.

## Author Contributions

**Selin Kalkan:** conceptualization (equal), data curation (equal), formal analysis (equal), investigation (equal), methodology (equal), supervision (equal), writing – original draft (equal), writing – review and editing (equal). **Kübra Incekara:** conceptualization (equal), formal analysis (equal), methodology (equal), software (equal), validation (equal), visualization (equal), writing – original draft (equal). **Mustafa Remzi Otağ:** data curation (equal), formal analysis (equal), methodology (equal), software (equal), writing – original draft (equal). **Emel Unal Turhan:** conceptualization (equal), investigation (equal), methodology (equal), writing – original draft (equal), writing – review and editing (equal).

## Disclosure

The authors did not use AI tools for any activities in this manuscript.

## Ethics Statement

This study does not involve any human or animal testing.

## Consent

Written informed consent was obtained from all study participants.

## Conflicts of Interest

The authors declare no conflicts of interest.

## Data Availability

Data will be made available on request.
